# Machine Learning-Aided Drug Repurposing for Screening COX-2 Inhibitors from Traditional Chinese Medicines

**DOI:** 10.3390/ph19060878

**Published:** 2026-05-31

**Authors:** Zhi-Xian Zhu, Bin Liu, Yi-Wen Xiao, Jun Chang

**Affiliations:** 1 School of Artificial Intelligence and Information Engineering, East China University of Technology, Nanchang 330013, China; rechuwureluo@outlook.com; 2 Key Laboratory of Natural Microbial Medicine Research of Jiangxi Province, School of Life Science, Jiangxi Science and Technology Normal University, Nanchang 330013, China; lb201001@126.com

**Keywords:** Random Forest Classifier, deep learning, graph neural network, COX-2 inhibitor, Traditional Chinese Medicine

## Abstract

**Background/Objectives**: Machine learning has emerged as a transformative force in drug discovery, revolutionizing traditional research paradigms and profoundly improving the efficiency, cost-effectiveness, and speed of the drug development cycle for novel drugs. Colorectal cancer is one of the most prevalent malignant tumors and imposes a heavy burden on global public health due to its high morbidity, mortality, and poor prognosis. Cyclooxygenase-2 (COX-2) is a key therapeutic target of colorectal cancer and has been extensively applied in the development of novel anti-colorectal cancer drugs. **Methods**: In this study, we systematically compared the performance of Random Forest Classifier (RFC), deep learning (DL), and graph neural network (GNN) models, including GAT (Graph Attention Network), GCN (Graph Convolutional Network), and MPNN (Message Passing Neural Network), with diverse features in the classification task of COX-2 inhibitors, based on a custom COX-2 inhibitors dataset and a Traditional Chinese Medicine (TCM)-derived compound library. The optimal model was subsequently used to screen for potential COX-2 inhibitors. Additionally, the key substructures governing COX-2 inhibitory activity were also identified and analyzed. Finally, the prioritized candidate compounds underwent experimental validation. **Results**: Both RFC and DL models outperformed GNN models. Through further comparative analysis of models’ predictive performance, the RFC model was ultimately verified as the optimal model for activity screening of TCM-derived compounds. The molecular interactions and binding affinities between predicted candidate compounds and COX-2 were further investigated. Finally, the selected lead compound, dehydrocostus lactone, was experimentally confirmed to possess potent COX-2 inhibitory activity. **Conclusions**: This study highlights that the RFC model is highly effective in screening bioactive components from TCM under small-dataset conditions, providing a solid foundation for subsequent related research in this field.

## 1. Introduction

Colorectal cancer (CRC) is the third most prevalent malignant tumor and the second leading cause of cancer-related deaths [1]. It imposes an enormous burden on public health due to its high morbidity and poor prognosis [2]. Nearly half of CRC patients suffer from incurable recurrence [3]. COX-2 has been used as a key target for CRC drug discovery due to its close correlation with CRC progression [4,5]. Traditional Chinese Medicine (TCM), a valuable reservoir of natural products [6], has emerged as a promising candidate for CRC drug development, with multi-target, multi-pathway regulatory effects and low toxicity [5]. These unique characteristics of TCM effectively complement the limitations of conventional single-target drugs. To date, several CRC inhibitors, including curcumin [6] from *Curcuma longa*, berberine [7] from *Coptis chinensis*, ginsenoside Rg5 [8] from *Panax ginseng*, and total saponins [9] from *Astragalus membranaceus*, have been successfully extracted from TCM herbs. However, the isolation of active ingredients from TCM remains challenging [10], primarily due to its complex chemical composition and diverse mechanisms of action (MOAs).

Machine learning (ML) is rapidly revolutionizing drug discovery, particularly in the repurposing and screening of TCM-derived bioactive components. Endowed with exceptional data-processing and pattern recognition capabilities, ML excels at analyzing large-scale datasets and establishing quantitative structure–activity relationship models. Therefore, ML offers a highly promising solution to the bottlenecks of traditional TCM research [11]. This unique advantage makes ML ideal for the rapid screening and repurposing of bioactive molecules from TCMs, as it enhances efficiency and unlocks their therapeutic potentials [12,13].

Within the ML landscape, deep learning (DL), graph convolutional networks (GNNs), and classic models such as the Random Forest Classifier (RFC) play distinct roles in molecular modeling [14,15], each with unique characteristics that make them well-suited for different research scenarios. DL is particularly adept at unraveling intricate patterns embedded in large-scale datasets through backpropagation [16], rendering it ideal for analyzing complex, high-dimensional molecular data. GNNs, by representing molecules as graphs (where nodes represent atoms and edges denote chemical bonds), can efficiently capture detailed molecular architectures [17]. This is a crucial capability for GNNs to understand the structural basis of molecular activity. Both DL and GNNs have been extensively employed in drug discovery [18,19,20], demonstrating their robustness in handling complex data and identifying subtle molecular patterns.

In contrast, classic models such as RFC serve a different role in TCM-derived drug discovery. RFC is characterized by its simplicity and efficiency in processing sparse or small-scale molecular datasets [21]. RFC offers stable performance without requiring substantial computational resources [22] and provides high interpretability through feature importance scores, which are critical for elucidating the MOAs of TCM-derived compounds. This stands in sharp contrast to DL and GNNs, which require more computational resources and longer training durations [23,24]. Their performance is highly dependent on hyperparameter tuning. The straightforward framework and strong interpretability of RFC make it a valuable tool in scenarios where computational resources are limited or where clear insights into molecular mechanisms are required. Both categories of models (advanced models such as DL and GNNs and classic models such as RFC) have been validated in relevant studies, including those focusing on inhibitor discovery for rheumatoid arthritis [25], P-glycoprotein [26], and Alzheimer’s disease [27].

**Figure 1:**
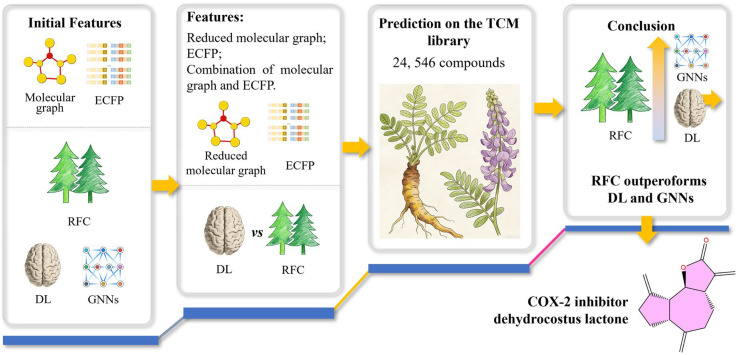
The architecture of the performance comparisons of models. ECFP, extended connectivity fingerprint; RFC, Random Forest Classifier; DL, deep learning; GNN, graph convolutional network. TCM, traditional Chinese medicine.

In the present study, we systematically compare the performance of RFC, DL, and GNNs, each combined with multiple molecular representations (ECFP, molecular graph, and their combinations), on a custom dataset of COX-2 inhibitors and a TCM library to predict COX-2 inhibitors from TCMs (Figure 1). The optimal model identified from this comparison was then used to screen a series of active COX-2 inhibitors, one of which was experimentally validated. This work not only explores the applicability of various ML models on TCM-derived drug screening but also provides a cost-effective and interpretable framework for the discovery of novel COX-2 inhibitors from TCM. Importantly, it highlights that the RFC model is highly effective for screening bioactive components from TCM using a small training dataset and can even outperform DL and GNNs in certain cases, thereby laying a foundation for future research in this field.

## 2. Results

### 2.1. Performance Comparison of the RFC, DL, GAT, GCN, and MPNN Models

Previous studies have demonstrated that ECFP outperforms both molecular descriptors and MACCkeys in feature effectiveness, while RFC outperforms other conventional ML models [25,28]. Accordingly, this study focuses on evaluating models using ECFP fingerprints, molecular graphs, and their concatenated features. Consequently, seven models were constructed for performance evaluations on the target dataset, each with clearly defined feature configurations: (1) RFC_ECFP (RFC with ECFPs as input); (2) DL_ECFP (DL with ECFPs as input); (3) RFC_graph (RFC with molecular graphs as input); (4) DL_graph (DL with molecular graphs as input); (5) GAT; (6) GCN; and (7) MPNN. The three GNN models (GAT, CCN, and MPNN) used molecular graphs exclusively as input features.

**Figure 2:**
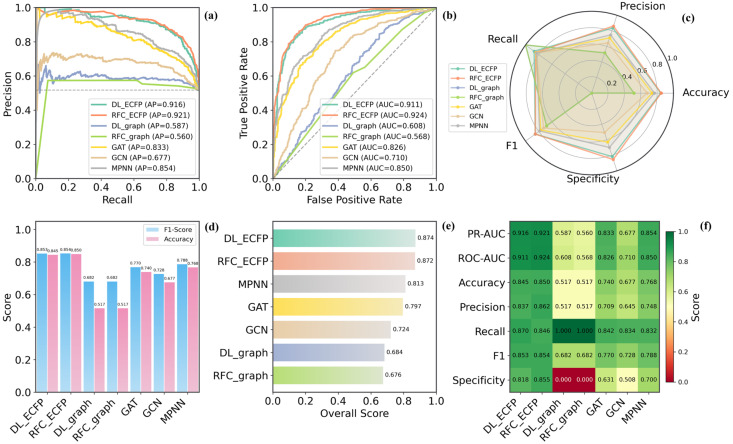
Comprehensive comparison of DL_ECFP, RFC_ECFP, DL_graph, RFC_graph, GAT, GCN, and MPNN models. (**a**) Precision–recall curves. (**b**) The receiver operating characteristic (ROC) curves. (**c**) The performance radar chart illustrates recall, precision, accuracy, specificity, and F1-score. (**d**) The F1-Score vs. accuracy correlation plot. (**e**) The model ranking chart. (**f**) The metrics heatmap of the aforementioned models. The overall score was calculated using the formula: Overall score = 0.3 × PR-AUC + 0.3 × F1-score + 0.2 × Accuracy + 0.2 × Recall. Specificity = TN/(TN + FP), where TN and FP represent true negatives and false positives, respectively. Ten-fold cross-validation with 10 repeated runs was conducted for the metrics (ROC-AUC, PR-AUC, accuracy, F1-score), and 95% confidence intervals were calculated via the bootstrap method (1000 resamples). All metrics are reported as mean ± 95% confidence interval.

As illustrated in Figure 2, RFC_ECFP and DL_ECFP outperformed the other five models, achieving the highest Average Precision (AP) values of 0.921 and 0.916, respectively (Figure 2a), as well as the highest Area Under the Curve (AUC) values of 0.924 and 0.911, respectively (Figure 2b). Both metrics are critical for evaluating these classification models on datasets: AP reflects the model’s ability to identify positive COX-2 inhibitors while minimizing false positive predictions, and AUC quantifies the overall discriminative power between active and inactive compounds.

Among the GNN models, a clear performance hierarchy was observed. MPNN performed best among the three models, with AP and AUC values reaching 0.854 and 0.850, respectively. This superior performance may be attributed to its inherent ability to effectively capture global molecular structural information through message passing between adjacent atoms in the molecular graph. GAT ranked second, achieving AP and AUC values of 0.833 and 0.826, respectively, benefiting from its attention mechanism, which selectively highlights key structural substructures critical for COX-2 inhibitory activity. In contrast, GCN exhibited a relatively worse performance, with AP and AUC values of only 0.677 and 0.710, respectively.

The advantages of RFC_ECFP and DL_ECFP extended beyond AP and AUC. They also demonstrated outstanding performances in F1-score, accuracy, and recall (Figure 2c,f). These complementary metrics further confirm the robustness of the two models: the F1-score balances precision and recall, accuracy reflects overall classification correctness, and recall ensures the model’s ability to capture potential active COX-2 inhibitors. Notably, RFC_graph and DL_graph exhibited abnormal performances: both models yielded a specificity of 0 (Figure 2f) and an accuracy of 0.517 (Figure 2d), which is close to the random guessing accuracy (0.50) of the test set. Detailed analysis revealed that these graph-based models misclassified all test compounds as active molecules. This severe classification bias may arise from the mismatch between their feature extraction mechanisms and the key structural features of COX-2 inhibitors, further exacerbated by insufficient feature learning due to dataset limitations and ultimately intensified by inadequate feature abstraction caused by architectural constraints. Collectively, these drawbacks render these graph-based models unsuitable for practical application in the COX-2 inhibitor classification task.

To address potential label noise from non-human-derived negative samples, a sensitivity analysis was conducted by restricting the negative dataset to human-derived non-inhibitors. The results showed that the RFC_ECFP still outperformed the DL_ECFP and MPNN models (Table S1, Supplement S1) across all key evaluation metrics (AUC: 0.91 vs. 0.89 for DL and 0.70 for MPNN; F1-score: 0.86 vs. 0.86 for DL and 0.77 for MPNN; accuracy: 0.83 vs. 0.84 for DL and 0.65 for MPNN), although the overall performance of all models slightly decreased due to the reduced sample size.

To comprehensively assess the aggregate performance of each model, an integrated score (Figure 2e) was calculated by combining key metrics, including PR-AUC, F1-score, accuracy, and recall. DL_ECFP achieved the highest overall score, followed closely by RFC_ECFP, whose score (0.872) was only 0.2% lower than DL_ECFP. The remaining models ranked as follows: MPNN (0.813), GAT (0.797), GCN (0.724), DL_graph (0.684), and RFC_graph (0.676).

In summary, RFC_ECFP and DL_ECFP demonstrated superior and well-balanced performance across all key evaluation metrics. Given their remarkable predictive performance in COX-2 inhibitor classification, RFC and DL were selected as the optimal model candidates for a more in-depth performance comparison.

### 2.2. Performance Comparison Between RFC and DL

Redundant features not only increase the computational complexity of ML algorithms but also induce adverse effects, such as training-set overfitting and poor test-set generalization [29]. To comprehensively compare the performance of RFC and DL models and to improve the performances of RFC graph and DL_graph, the Boruta module [30], integrated with RFC, was employed to identify and eliminate redundant features in molecular graphs. Specifically, the RFC was configured with 1000 decision trees and no maximum depth constraint, ensuring that the model could fully capture the complex intrinsic patterns embedded in molecular graphs. For the Boruta algorithm, the number of iterations was automatically determined, and a significance level of 0.05 was set to assess the feature importance.

To reduce the redundancy of the ECFP, a variance threshold (1 × 10^−5^) and a Pearson correlation coefficient threshold of 0.95 were employed to remove low-variance and highly correlated features, respectively. This preprocessing step was intended to improve the efficiency and effectiveness of subsequent model training. As a result, the dimensionality of graph features was reduced from 258 to 41, while the dimensionality of ECFP was reduced from 2048 to 2036.

Subsequently, six models were constructed for systematic performance evaluations, with each model adopting specific feature configurations: (1) DL_ECFP_r, a DL model using dimensionality-reduced ECFPs; (2) DL_graph_r, a DL model using dimensionality-reduced molecular graphs; (3) DL_ECFP_graph, a DL model using combination of ECFPs and molecular graphs; (4) RFC_ECFP_r, an RFC model using dimensionality-reduced ECFPs; (5) RFC_graph_r, an RFC model using dimensionality-reduced molecular graphs; and (6) RFC_ECFP_graph, an RFC model using a combination of ECFPs and molecular graphs.

**Figure 3:**
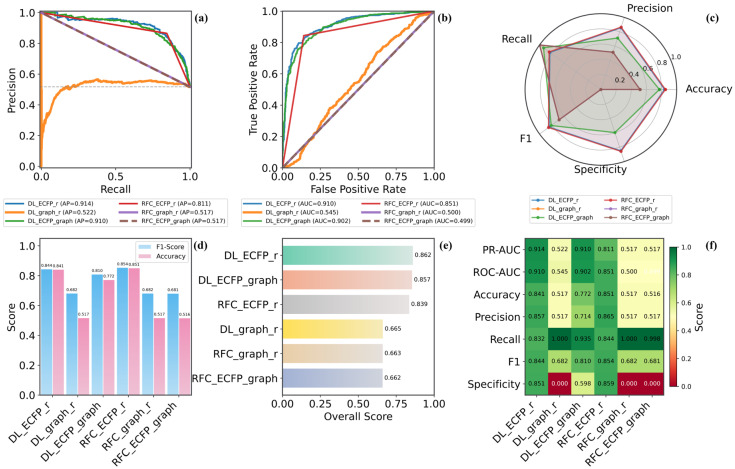
Comprehensive performance comparison among DL_ECFP_r, DL_graph_r, DL_ECFP_graph, RFC_ECFP_r, RFC_graph_r, and RFC_ECFP_graph. (**a**) Precision–recall curves; (**b**) ROC curves; (**c**) performance radar chart illustrating recall, precision, accuracy, specificity, and F1-score; (**d**) correlation plot between F1-score and accuracy; (**e**) model performance ranking chart; (**f**) performance metrics heatmap of the investigated models. Notably, the curves of RFC_graph_r and RFC_ECFP_graph overlap completely in panels (a) and (b).

As illustrated in Figure 3, models using ECFP-based features (DL_ECFP_r, DL_ECFP_graph, and RFC_ECFP_r) significantly outperformed those using graph-based features. Specifically, these ECFP-based models achieved the highest AP values of 0.914, 0.910, and 0.811 (Figure 3a), and the highest AUC values of 0.910, 0.902, and 0.851 (Figure 3b). In contrast, models with graph-based features (DL_graph_r, RFC_graph_r, and RFC_ECFP_graph) yielded AP and AUC values ranging from 0.499 to 0.545, which are close to random guessing. This finding further confirms that ECFP-based representations are critical for achieving satisfactory classification performance, whereas graph-based features, even after dimensionality reduction, fail to enhance the discriminative ability.

Consistent with the trends observed in AP and AUC, F1 scores, accuracy (Figure 3d), recall, and precision for DL_ECFP_r, DL_ECFP_graph, and RFC_ECFP_r remained at high levels (Figure 3c,f). Similar to the original graph-based models, both RFC_graph_r and DL_graph_r misclassified all test compounds as active, resulting in a specificity of 0 and a recall of 1. The overall performance scores (Figure 3e) further verify that ECFP-based RFC and DL models achieve consistently excellent performance, reinforcing that ECFP-based input delivers robust, superior performance across both RFC and DL architectures for this classification task.

### 2.3. Predictive Behavior of RFC_ECFP and DL_ECFP on Herb Dataset

**Figure 4:**
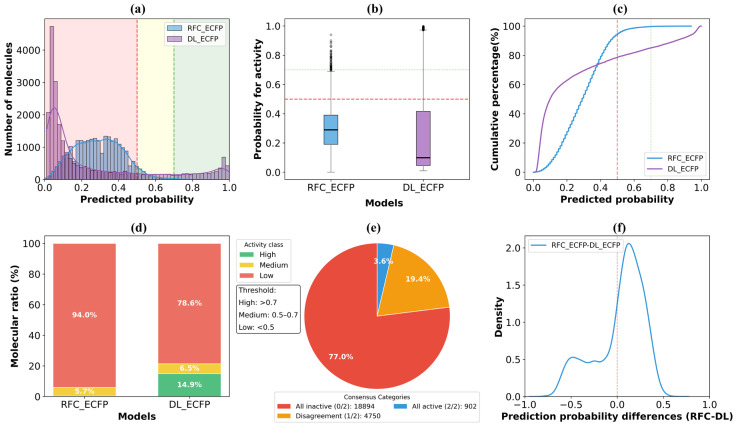
Comparative generalization performance analysis between RFC_ECFP and DL_ECFP models on TCM-derived molecule activity prediction. (**a**) Distribution of predicted activity probabilities of RFC_ECFP and DL_ECFP. The red dashed line indicates the activity decision threshold of 0.5, and the green dashed line represents the high-activity threshold of 0.7. The red shaded region, yellow shaded region, and green shaded region correspond to low-activity (<0.5), medium-activity (0.5–0.7), and high-activity (>0.7) molecules, respectively. (**b**) Comparison of overall predicted probability distributions between two models. (**c**) Cumulative distribution of model-predicted probabilities. (**d**) Comparison of molecular ratios in high-, medium-, and low-activity categories predicted by different models. RFC_ECFP contains 0.3% high-activity molecules, which is not shown in the bar chart because of its extremely low proportion. (**e**) Consensus and disagreement distribution of prediction results between RFC_ECFP and DL_ECFP. (**f**) Distribution of prediction probability differences (RFC_ECFP − DL_ECFP).

To assess the predictive capabilities of the ECFP-based RFC and DL models, an independent TCM library was used to evaluate their classification performance. Comprehensive comparative analysis (Figure 4a–f) systematically reveals substantial divergence in their predictive behavior on this novel chemical space, directly uncovering critical deficiencies in predictive calibration. As illustrated in Figure 4d, at a standard probability threshold of 0.5, DL_ECFP predicted implausibly high proportions of active compounds (21.4% of the TCM library), which is starkly inconsistent with established pharmacological knowledge regarding realistic hit rates in drug discovery. In contrast, RFC_ECFP yielded a chemically plausible and conservative prediction, with only 6.0% of compounds classified as active inhibitors. This pattern of pronounced overconfidence in DL_ECFP persisted even at a stringent cutoff of 0.7 (14.9% of the TCM library; Figure 4d), whereas RFC_ECFP yielded only 5.7% of predictions as active, demonstrating its stringent, high-confidence screening behavior.

Consensus analysis (Figure 4e) further quantified this discrepancy, revealing that 19.4% of molecules exhibited a disagreement between the two models. The vast majority of these discordant cases were characterized by DL_ECFP assigning active labels while RFC_ECFP maintained inactive predictions, directly revealing DL_ECFP’s tendency to label inactive compounds as active erroneously. Furthermore, Figure 4f displays the distribution of prediction probability differences (RFC_ECFP − DL_ECFP). The density was strongly concentrated in the negative region, quantitatively confirming that DL_ECFP consistently yielded higher predicted probabilities than RFC_ECFP across most molecules. This result directly and intuitively demonstrates the systematic positive bias and inherent overconfident predictive behavior of DL_ECFP on small-scale TCM datasets.

**Figure 5:**
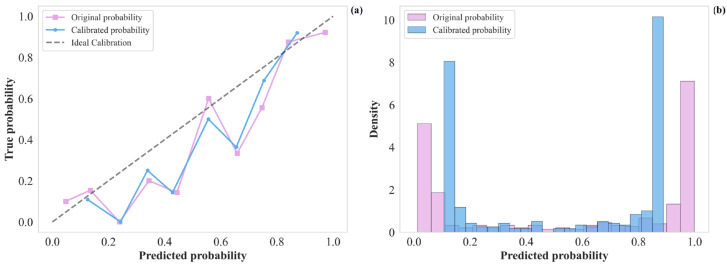
Calibration performance analysis of the DL_ECFP model on TCM molecular activity prediction. (**a**) Reliability diagram (calibration curve) of the original DL_ECFP and calibrated DL_ECFP model. The dashed line represents the ideal perfectly calibrated prediction. (**b**) Probability distribution comparison between the original predicted probability and the calibrated probability of DL_ECFP.

Given the overconfident predictive behavior and suboptimal calibration of DL_ECFP, a post-training probability calibration framework was implemented to assess whether calibrated probability outputs could rectify its unreliable predictions on the TCM library. As shown in Figure 5, calibration significantly improved the model’s probability metrics: the Brier score decreased from 0.1088 to 0.1075 (Figure 5a), and the model’s Expected Calibration Error (ECE) was reduced by 22.2%. Consequently, the number of positive predictions on the TCM library decreased from 5, 259 (21.4% of the TCMs) to 5, 132 (20.9% of the TCMs) (Figure 5b). Critically, even after calibration, the DL_ECFP model continued to predict a hit rate exceeding 20%, which remains biologically and chemically implausible for a novel compound library and stands in stark contrast to the conservative estimate of 6% from RFC_ECFP. This finding indicates that the overconfidence of the DL_ECFP model represents a fundamental, systemic issue intrinsic to its underlying learning architecture, rather than a superficial miscalibration bias that can be fully rectified via post hoc adjustments.

Detailed probability distribution analysis (Figure 4a–c) further elucidated the mechanistic underpinnings of this divergence. RFC_ECFP adopted a conservative, uncertainty-aware strategy: it assigned higher mean probabilities to compounds in the inactive region, indicating greater hesitation when making definitive negative class assignments, while providing more moderate, cautious probability estimates for the active region. In contrast, DL_ECFP exhibited a pronounced positive bias, with its predicted probability distribution (Figure 4a) and cumulative curve (Figure 4c) shifted significantly toward higher values, and a higher median probability (Figure 4b), reflecting its inherent overconfident predictive behavior.

Collectively, these results demonstrate that RFC_ECFP, with its chemically plausible and conservative predictive behavior, represents a reliable choice for repurposing herbal medicines, particularly given the implausibly high hit rates predicted by DL_ECFP (even after calibration) and the established pharmacological constraints of TCM compound screening.

### 2.4. Screeing Active COX-2 Inhibitors from TCMs

The predicted activities of RFC_ECFP were further employed to screen COX-2 inhibitors. Compounds with a prediction probability exceeding 0.75 were selected for molecular docking. Those with affinities below −6.5 kcal/mol were further subjected to binding affinity energy calculation (Table 1). Primin and indomethacin have been reported to be active COX-2 inhibitors [31,32]. Subsequently, lead compounds with binding affinity energy lower than that of tolfenamic acid (−35.4492 kcal/mol), the active ligand in the COX-2 crystal, were classified as potential COX-2 inhibitors. Consequently, eight compounds were selected for receptor–ligand interaction analysis: irisquinone (−50.7451 kcal/mol), pallasone B (−46.6878 kcal/mol), dehydrocostus lactone (−60.8297 kcal/mol), mexicanin E (−51.0447 kcal/mol), artecanin (−37.2687 kcal/mol), parthenolide (−53.2217 kcal/mol), 3-epizaluzanin C (−41.6978 kcal/mol), and 4β-methoxycostuslactone (−37.9408 kcal/mol).

**Figure 6:**
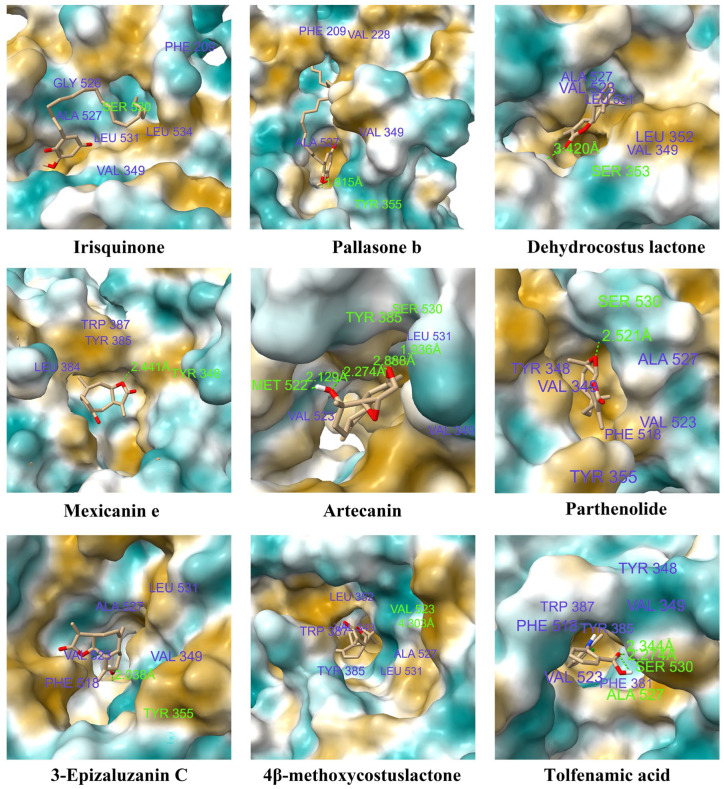
Three-dimensional binding conformations and non-covalent interaction diagrams between the COX-2 receptor (PDB ID: 5IKT) and candidate lead compounds. Green amino acid residues represent those involved in hydrogen bond interactions, while blue residues indicate residues with non-hydrogen bond interactions. To better visualize the binding conformations of ligands in the receptor’s active pocket, the interacting amino acid residues and detailed non-covalent interaction networks were shown, and the 3D structure of 5IKT was properly rotated, with surface representations of partially removed amino acid residues. Obvious differences in binding modes and interaction details among receptor–ligand complexes are evident in subpanels. The redocking of tolfenamic acid to 5IKT provided an RMSD of 0.4942 (Figure S1, Supplement S1).

**Figure 7:**
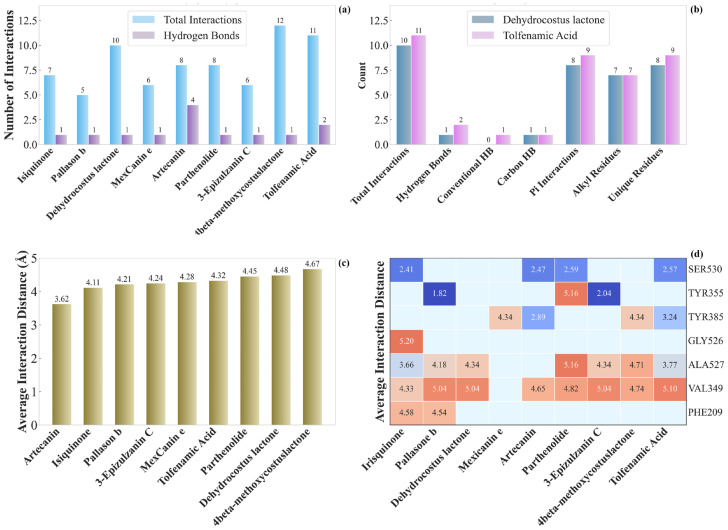
Statistical analysis of the interactions between lead compounds, positive controls, and the COX-2 receptor. (**a**) Statistics of total intermolecular interactions and hydrogen bond numbers for each compound binding to COX-2. (**b**) Classification comparison of different interaction types between dhydrocostus lactone and tolfenamic acid binding to COX-2. (**c**) Average binding interaction distance of each compound with the COX-2 receptor. (**d**) Heatmap of average interaction distance between individual amino acid residues of the COX-2 and each tested compound.

Figure 6 illustrates the three-dimensional binding conformations and detailed non-covalent interaction networks of candidate lead compounds with the COX-2 receptor (PDB ID: 5IKT), thereby intuitively demonstrating their potential COX-2 inhibitory activities. To better characterize these binding patterns, statistical analysis of intermolecular interactions was further performed (Figure 7). 4β-methoxycostuslactone exhibited the highest number of non-hydrogen bond interactions, followed by tolfenamic acid and dehydrocostus lactone (Figure 7a). In contrast, tolfenamic acid formed two hydrogen bonds with bond lengths of 2.34 Å and 2.28 Å. Further interaction analysis (Figure 7b) revealed that dehydrocostus lactone exhibits a highly similar interaction pattern (hydrogen bonds, Pi interactions, alkyl residues interactions) to the positive control tolfenamic acid when binding to the COX-2 active pocket. The total interactions between dehydrocostus lactone and 5IKT were similar to those of tolfenamic acid (Figure 7a), with favorable average interaction distances (Figure 7c,d). Detailed information regarding the interactions between the lead compounds and the 5IKT receptor is provided in Supplement S2. Ultimately, based on a comprehensive analysis of the interaction profiles and an evaluation of the market availability of these lead compounds, dehydrocostus lactone was selected for experimental verification of its COX-2 inhibitory activity.

### 2.5. Key Substructures for COX-2 Inhibitory Activity

**Figure 8:**
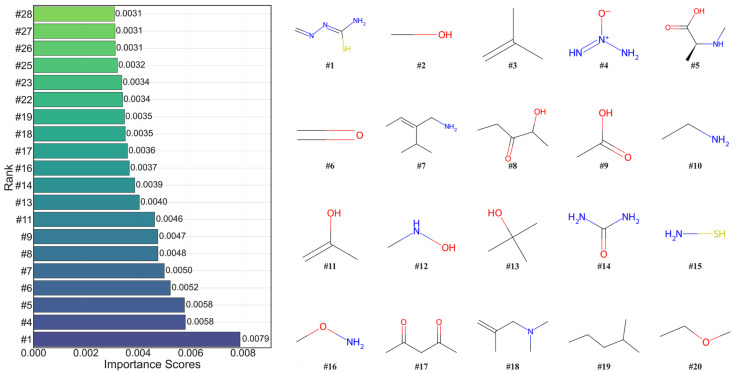
The importance of substructures for RFC_ECFP prediction.

As illustrated in Figure 8, the top 20 key functional substructures for COX-2 inhibitory activity were ranked by the interpretable RFC_ECFP model. These high-influence substructures are abundant in nitrogen-containing functional groups, oxygen-containing polar groups, unsaturated bonds, and sulfur-containing moieties, underscoring their crucial role in mediating the COX-2 inhibitory activity of herbal molecules. Specifically, nitrogen-containing heteroatom functional groups dominate the top-ranked beneficial substructures, which are well documented to mediate key receptor-ligand interactions with COX-2. They act as versatile hydrogen-bond donors or acceptors, forming stable interactions with polar amino acid residues in the active site, and also participate in salt bridges with positively charged residues or in hydrophobic stacking with aromatic residues. Meanwhile, hydroxyl/carbonyl oxygen groups, unsaturated alkene/carbonyl structures, and thiol groups also contribute substantially to the binding activity, as shown in the visualized top substructure skeletons.

These interactions are likely to enhance the binding affinities and specificity between the inhibitors and COX-2, which may contribute to the molecule’s inhibitory potency. This insight, enabled by the interpretability of the RFC_ECFP, further supports the statistical relevance of the identified substructures to COX-2 inhibitory activity. It highlights the model’s unique value in bridging structural features to functional activity through potential molecular interactions, while acknowledging that additional experimental or structural validation is required to confirm the direct mechanistic role of these substructures.

### 2.6. Inhibitory Activity of Dehydrocostus Lactone

**Figure 9:**
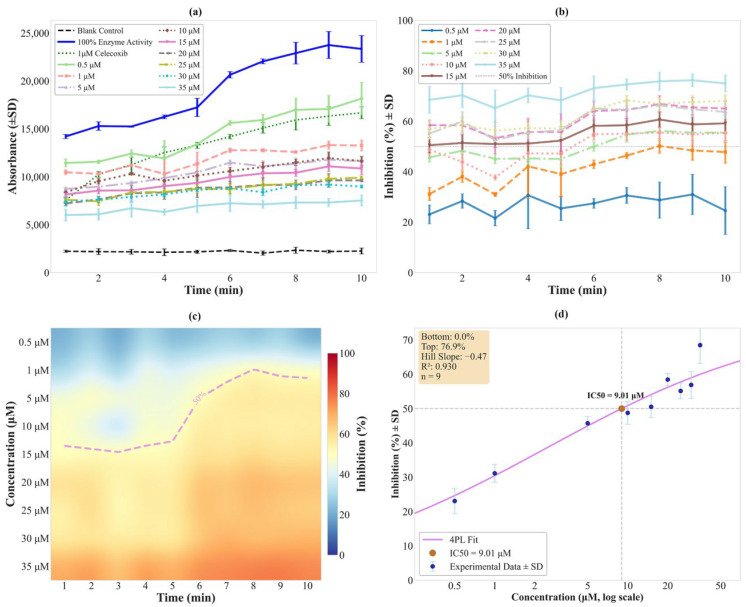
Experimental COX-2 inhibitory activity assay of dehydrocostus lactone. (**a**) Time-dependent absorbance change curves of dehydrocostus lactone at different concentrations, along with blank control and celecoxib positive control. (**b**) Time-dependent COX-2 inhibition percentage curves of dehydrocostus lactone at serial concentrations. (**c**) Heatmap visualization of COX-2 inhibition percentage under different concentrations and time conditions. (**d**) Four-parameter logistic (4PL) fitting curve for IC_50_ calculation of dehydrocostus lactone against COX-2.

The COX-2 inhibitory activity of dehydrocostus lactone was evaluated using an in vitro enzymatic inhibition assay at serial concentrations of 0.5, 1, 5, 10, 15, 20, 25, 30, and 35 μM. Absorbance signals were continuously monitored at 1 min intervals over a 10 min reaction period, along with blank and celecoxib-positive control groups. As illustrated in Figure 9a,b, the inhibition rate at each concentration increased over time throughout the detection period. It reached a stable plateau at 10 min. The concentration–time inhibition heatmap (Figure 9c) further intuitively demonstrated its dose-dependent inhibitory effect, revealing approximately 50% COX-2 inhibition observed at 5 μM after 6 min of incubation. A four-parameter logistic (4PL) nonlinear regression model was employed to fit the dose–response curve at 1 min (Figure 9d), with fitted parameters: Bottom = 0%, Top = 76.9%, Hill slope = −0.47, and R^2^ = 0.93. Notably, the fitted maximum inhibition (Top) value deviated from the theoretical 100% inhibition level. Accordingly, the IC_50_ of dehydrocostus lactone was defined as the experimental concentration corresponding to the actual 50% inhibition level instead of the default fitted curve output, and the final determined IC_50_ value was 9.01 μM.

## 3. Discussion

DL and GNN have been widely employed in drug discovery due to their remarkable capabilities to capture complex patterns from large-scale datasets [16] and to model intricate molecular structures [17], respectively. These advanced data-driven models have attracted considerable attention in recent years and have been integrated into nearly all stages of modern drug discovery pipelines, including target identification, virtual screening, molecular property prediction, and drug repurposing [33]. In contrast, classic ML models such as RFC have long been overlooked and marginalized in mainstream research [34]. Nevertheless, classic tree-based ensemble models possess inherent and irreplaceable advantages, including extremely low computational resource consumption, convenient deployment, straightforward implementation with minimal hyperparameter tuning, and excellent intrinsic interpretability based on feature-importance scores [34]. These practical characteristics render RFC particularly valuable for translational and practical drug discovery applications, especially in resource-constrained laboratory settings where high-performance computing facilities are unavailable.

In this study, we systematically compared the performance of RFC, DL, and GNN models in repurposing TCM-derived drugs. Our comparative results reveal that RFC achieves more stable and favorable predictive performances than DL and GNNs under small-data scenarios. Notably, DL models inherently rely on large-scale training data and robust feature representations to optimize parameters. When applied to new datasets (such as the TCM compound library used in this study), DL models tend to exhibit overconfidence in their predictions, a phenomenon that is not solely attributed to the model architecture itself but also influenced by multiple contributing factors, including label noise, potential scaffold leakage, dataset imbalance, insufficient hyperparameter optimization, and limitations in molecular representation. This overconfidence substantially increases the false positive rate and results in a high proportion of molecules erroneously classified as active candidates [35]. Accordingly, the prediction ability and practical reliability of DL models under small-data conditions require more cautious evaluation and validation. More importantly, even after applying standard external probability calibration, the intrinsic overconfident bias in the DL models cannot be effectively eliminated, and their predictive accuracy still fails to meet the rigorous criteria for reliable virtual screening. This phenomenon is highly consistent with previously reported findings in molecular property prediction research [36]. The underlying mechanism lies in the overparameterized structure of neural networks, which can easily memorize noise and outliers in small datasets rather than learning generalized structure–activity relationships, leading to biased and overestimated active probabilities. By comparison, RFC employs an ensemble decision mechanism comprising multiple independent decision trees, which effectively mitigates both overfitting and overconfidence without requiring large-scale training data.

In general, datasets containing data from multiple species tend to compromise model performance. However, the RFC_ECFP exhibited remarkable robustness, compared with the RFC_ECFP model trained on a training set containing human-only negative inhibitors (Table S1, Supplement S1). This observation highlights the unique advantage of RFC_ECFP in handling heterogeneous datasets. Furthermore, the performance rankings of the human-only-data-trained RFC_ECFP, DL_ECFP, and MPNN models remained consistent with those of their multi-species-data-trained counterparts. This consistency can be attributed to the fact that the core performance differences among these three models arise from their inherent structural characteristics and data dependency, rather than the species origin of the negative samples. The ensemble decision-making mechanism of RFC_ECFP enables it to focus on the intrinsic structure–activity relationships of molecules, rendering its performance less susceptible to interference from the species diversity of negative samples. In addition, the interpretability of RFC_ECFP, based on feature importance, ensures the reliable identification of key molecular features associated with activity, which is not affected by the species information of negative samples. Based on the above analysis, in research on the application of RFC_ECFP to TCM drug repurposing, integrating activity data across species to expand the training dataset is both feasible and beneficial. This integration allows the RFC model to learn a more diverse set of active molecular structures, thereby further enhancing its predictive capacity in TCM-derived drug repurposing tasks.

Predictive performance of these models is highly dependent on dataset-splitting strategies, primarily due to scaffold overlap between training and test sets under random partitioning. When the random-split-trained models were applied to the scaffold-split test set, the predictive performance of the RFC_ECFP and DL_ECFP models remained relatively stable (Table S2, Supplement S1). In contrast, the MPNN model suffered significant performance deterioration. When models were retrained and evaluated on the scaffold-split datasets, all the three models exhibited some decline in performance (Table S3, Supplement S1), confirming that random splitting introduces scaffold leakage and yields relatively optimistic estimates. Nevertheless, the models’ performance rankings remained unchanged.

Although random splitting bears the risk of scaffold leakage, these results support a critical conclusion: the random-splitting strategy enables the RFC_ECFP model to better identify bioactive molecules with identical or similar core scaffolds but distinct substituents. Random splitting is therefore a suitable choice for modelling in early-stage drug discovery, where identifying structural analogues with potential bioactivity is a core requirement. From a practical application perspective, random splitting maintains reasonable structural continuity between training and test compounds, allowing the RFC_ECFP model to effectively capture activity patterns of bioactive molecules sharing identical or similar core scaffolds.

Considering RFC’s advantages of low computational cost, simple hyperparameter optimization process, favorable interpretability, and stable performance on small-scale sets, it can be concluded that RFC represents a promising and relatively more suitable option for small-scale classification tasks aiming at distinguishing active and inactive molecules, such as virtual screening and identification of COX-2 inhibitors from TCM compounds. Furthermore, the findings of this study provide practical guidance for model selection in natural product drug discovery. Instead of unthinkingly pursuing advanced DL architectures, researchers should select appropriate algorithms based on dataset scale, computational resources, and interpretability requirements. For small-sample TCM molecule screening projects, RFC is not only an efficient alternative but also a more reliable and translatable choice than complex models such as DL and GNNs. Meanwhile, this work does not deny the advantages of DL and GNNs in large-data scenarios. Instead, it highlights the importance of matching model complexity to dataset scale to avoid misleading false positive results in real-world drug screening.

## 4. Materials and Methods

### 4.1. Datasets

The COX-2 inhibition datasets were retrieved from the ChEMBL database (https://www.ebi.ac.uk/chembl/, accessed on 22 October 2025), covering six species of *Homo sapiens* (CHEMBL230), *Rattus norvegicus* (CHEMBL2977), *Bos taurus* (CHEMBL3331), *Canis lupus familiaris* (CHEMBL4033), *Ovis aries* (CHEMBL4102), and *Mus musculus* (CHEMBL4321). Only entries with available IC_50_, inhibition, INH, and K_i_ values were retained. A unified preprocessing pipeline was applied to all retained entries. Briefly, the SMILES strings for all compounds were standardized using the RDKit toolkit [37] to ensure consistent structural representations. For duplicate records with identical activity units, the mean activity value was calculated, and a single unique record was retained, with redundant entries removed. For duplicate records reported in different assays, only compounds with consistent activity labels (active or inactive) across all assays, as defined in Table 2, were preserved and duplicated. Those entries with contradictory activity annotations were discarded. 

Following preprocessing, compounds from *Homo sapiens* were classified into inhibitors and non-inhibitors based on the criteria in Table 2. To construct a balanced dataset, additional non-inhibitors were collected from the preprocessed datasets for the five non-human species listed above, in accordance with the classification criteria in Table 2.

The final COX-2 inhibition dataset comprised 2627 inhibitors and 2446 non-inhibitors. This integrated dataset was then randomly split into training and test sets, with the test set accounting for 20% of all compounds (Supplements S3 and S4). To assess the predictive behaviors of the subsequent models and explore the potential of TCM repurposing for COX-2 inhibition, a TCM compound library was compiled from the HERB database (http://herb.ac.cn/, accessed on 23 October 2025). Only organic compounds possessing valid SMILES strings and absent from the COX-2 training dataset were preserved. Ultimately, 24,546 TCM-derived compounds were obtained (Supplement S5).

### 4.2. Models

**Figure 10:**
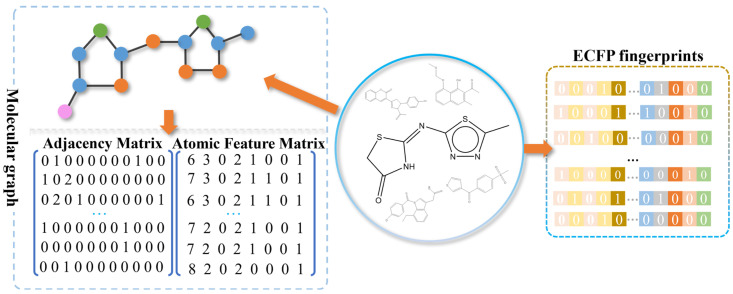
Conversion of molecular structures to molecular graphs; ECFP fingerprints.

Molecules were represented as SMILES strings and subsequently converted into features: ECFP (radius = 2, number of bits = 2048), molecular graphs (nodes represent atoms, and edges denote chemical bonds), and their concatenation. For each atom in a molecule, eight structural and electronic attributes were extracted, including atomic number, atomic degree, formal charge, atomic hybridization state, aromaticity (1 for aromatic atom and 0 for non-aromatic atoms), number of bonded hydrogen atoms, number of radical electrons, and ring membership (1 for atom in a ring and 0 otherwise) (Figure 10). All feature calculations were performed using the RDKit toolkit [37]. To evaluate the performance of RFC, DL, and GNN models (GAT, GCN, and MPNN), each was trained on ECFP fingerprints, molecular graphs, and their concatenation. The codes for all models, including all relevant parameters, are publicly available on the public GitHub repository (https://github.com/changjun8772/models_comparision, accessed on 23 October 2025). Their performances in compound classification and predictive behaviors were then compared on the COX-2 inhibitor dataset and the external TCM compound library.

### 4.3. Molecular Docking

Molecular docking was employed to evaluate the binding affinities between the predicted candidate hits and COX-2. The 3D crystal structure of human COX-2 [38] (PDB ID 5IKT; resolution 2.45Å; *Homo sapiens*) was retrieved from the PDB database (https://www.rcsb.org/, accessed on 27 October 2025). The docking pocket was defined based on the key active residues that interact with the co-crystallized ligand (tolfenamic acid), including LEU531, VAL349, VAL523, VAL116, ALA527, LEU352, TYR385, and SER530. Molecular docking was performed using Autodock vina 1.2. Ligand structures (SDF format) and the COX-2 receptor structure (PDB format) were converted into PDBQT format via the Open Drug Discovery Toolkit [39]. The docking grid parameters were configured as follows: center_x: 165.71, center_y: 186.268, center_z: 193.371, size_x: 26.96, size_y: 26.96, and size_z: 26.96. All remaining parameters were kept at default values. Binding free energy (ΔG_bind) was calculated using a custom Python 3.11 script that strictly replicates the AMBER 2023 mmgbsa workflow, adopting the ff14SB force field and GB2 model to compute ΔG_bind as the sum of molecular mechanics energy (ΔE_MM, including van der Waals and electrostatic terms) and solvation energy (ΔG_sol, including GB polar and SASA non-polar terms), consistent with the default settings of AMBER MM-GBSA calculations.

### 4.4. COX-2 Inhibitory Assay

COX-2 inhibitory activity was determined using a COX-2 Inhibitor Screening Kit (Beyotime Biotechnology, Shanghai, China), according to the manufacturer’s instructions with minor modifications. Briefly, test samples were mixed with assay reagents and incubated at 28 °C for 10 min. After adding the COX-2 probe and substrate to the reaction mixture, the mixture was incubated at 37 °C for another 10 min. Fluorescence measurements were conducted at an excitation wavelength of 560 nm and an emission wavelength of 590 nm. Fluorescence intensity was recorded at 1 min intervals over a total duration of 10 min. The COX-2 inhibition rates were calculated using the following formula:

$$Inhibitory\;\left(\%\right)=\;100\%\times \frac{{RFU}_{100\%enzymatic\;activity}-{RFU}_{inhibitor}}{{RFU}_{100\%enzymatic\;activity}-{RFU}_{blank}}$$

where *RFU* denotes relative fluorescence unit, and all experiments were performed in triplicate. The IC_50_ of the inhibitor was calculated by fitting the kinetic inhibition data to a dose–response curve.

## Figures and Tables

**Table 1:** The lead compounds that were virtually screened from TCMs with affinities below −6.5 kcal/mol.

Ingredient Names	Binding Affinity Energy (kcal/mol)	Affinity(kcal/mol)
Tolfenamic acid	−35.4492	−8.3
Irisquinone	−50.7451	−8
Isodehydrocostus lactone	−30.6265	−7.9
Pallasone B	−46.6878	−7.9
Dehydrozaluzanin C	−9.5588	−7.7
Ermanthin	−10.943	−7.5
Zaluzanin D	−20.8173	−7.1
dehydrocolorlespdin	2.2108	−7
Dehydrocostus lactone	−60.8297	−7
Mexicanin E	−51.0447	−7
Primin [31]	−53.9939	−7
artecanin	−37.2687	−6.8
parthenolide	−53.2217	−6.8
3-Epizaluzanin C	−41.6978	−6.6
Indomethacin [32]	−48.756	−6.6
4β-methoxycostuslactone	−37.9408	−6.5

**Table 2:** The definition of positive and negative inhibitors.

Activity Type	Inhibitors	Non-Inhibitors
IC_50_, Ki	≤1000 nM	≥20,000 nM
Inhibition, INH	≥70%	≤50%
Number of entities	2627	2446

## Data Availability

The original contributions presented in this study are included in the article/Supplementary Material. Further inquiries can be directed to the corresponding authors.

## Supplementary Materials

The following supporting information can be downloaded at: https://www.mdpi.com/article/10.3390/ph19060878/s1. Supplement S1: Superposition of the co-crystallized tolfenamic acid and its redocked pose (Figure S1), performance comparison of RFC, MPNN, and DL models on the human-only negative dataset for sensitivity analysis (Table S1), performance comparison of randomly-split-trained RFC_ECFP, DL_ECFP, MPNN models on the scaffold-aware split test set (Table S2), and performance comparison of scaffold-split-trained RFC_ECFP, DL_ECFP, MPNN models on the scaffold-aware split test set (Table S3); Supplement S2: Interactions between lead compounds and 5IKT; Supplement S3: test dataset; Supplement S4: training dataset; Supplement S5: TCM ingredients.

## Abbreviations

The following abbreviations are used in this manuscript:

COX-2	Cyclooxygenase-2
RFC	Random Forest Classifier
DL	Deep learning
GNN	Graph neural network
GAT	Graph Attention Network
GCN	Graph Convolutional Network
MPNN	Message Passing Neural Network
TCM	Traditional Chinese Medicine
CRC	Colorectal cancer
ML	Machine learning
MOA	Mechanisms of action
ECFP	Extended connectivity fingerprint
RFC_ECFP	RFC with ECFPs as model’s input
DL_ECFP	DL with ECFPs as model’s input
RFC_graph	RFC with molecular graphs as model’s input
AP	Average Precision
AUC	The highest Area Under the Curve
ROC	The Receiver Operating Characteristic
TN	True negative
FP	False positive
DL_ECFP_r	DL model using dimensionality-reduced ECFP
DL_graph_r	DL model using dimensionality-reduced molecular graph
DL_ECFP_graph	DL model using combination of ECFP and molecular graph
RFC_ECFP_r	RFC model using dimensionality-reduced ECFP
RFC_graph_r	RFC model using dimensionality-reduced molecular graph
RFC_ECFP_graph	RFC model using a combination of ECFP and molecular graph
ECE	Expected Calibration Error
