# Supplementary material for: Machine Learning-Aided Drug Repurposing for Screening COX-2 Inhibitors from Traditional Chinese Medicines
Source: Pharmaceuticals (Basel). 2026 May 31;19(6):878. doi: 10.3390/ph19060878 (PMC13306183; doi:10.3390/ph19060878)
Supplement: Supplementary file 1 [file pharmaceuticals-19-00878-s001.zip › Supplement S1.pdf]

Table S1. Performance comparison of RFC, MPNN, and DL models on the human-only negative dataset for sensitivity analysis.

Models	precision	recall	F1	AUC	Accuracy
RFC	0.86	0.86	0.86	0.91	0.83
MPNN	0.64	0.96	0.77	0.70	0.65
DL	0.89	0.84	0.86	0.89	0.84

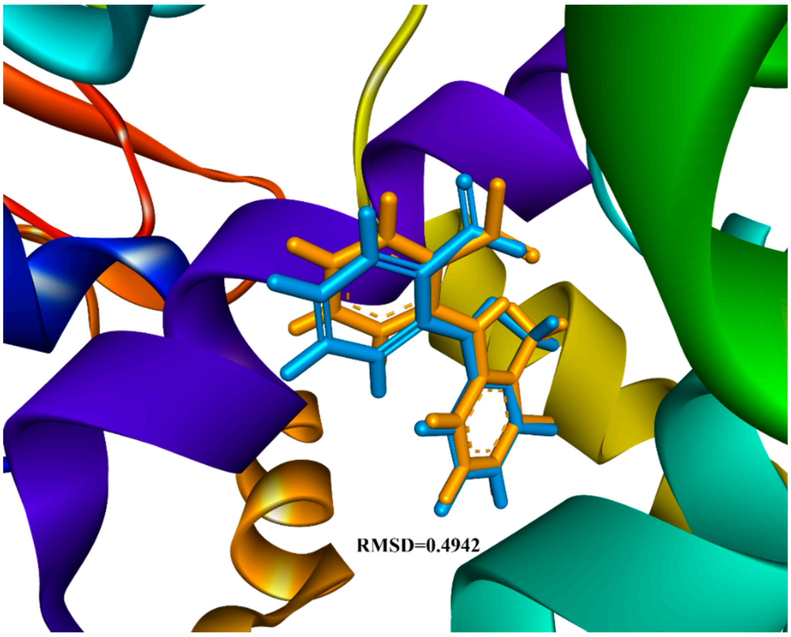

Figure S1. Superposition between the co-crystallized tolfenamic acid and its redocked pose. The blue molecule represents the native ligand (tolfenamic acid) from the COX-2 crystal structure (PDB: 5IKT), and the orange molecule corresponds to its redocked pose obtained using the molecular docking protocol.

Table S2. Performance comparison of randomly-split-trained RFC_ECFP, DL_ECFP, MPNN models on the scaffold-aware split test set.

Models	precision	recall	F1	AUC	Accuracy
RFC	0.87	0.97	0.92	0.98	0.91
MPNN	0.55	0.98	0.70	0.57	0.58
DL	0.84	0.95	0.89	0.95	0.88

Table S3. Performance comparison of scaffold-split-trained RFC_ECFP, DL_ECFP, MPNN models on the scaffold-aware split test set.

Models	precision	recall	F1	AUC	Accuracy
RFC	0.76	0.84	0.80	0.88	0.78
MPNN	0.70	0.81	0.75	0.75	0.73
DL	0.76	0.82	0.89	0.84	0.78
